# General health status of youth with autism with and without intellectual disabilities transitioning from special education, and its relationship to personal and family circumstances: longitudinal cohort study

**DOI:** 10.1080/20473869.2021.1966600

**Published:** 2021-08-30

**Authors:** Ewelina Rydzewska, Michael Fleming, Daniel Mackay, Genevieve Young-Southward, Jan Blacher, Yasamin Ross Bolourian, Keith Widaman, Sally-Ann Cooper

**Affiliations:** 1 Institute of Health and Wellbeing, University of Glasgow, Glasgow, UK; 2 Graduate School of Education, University of California Riverside, Riverside, CA, USA

**Keywords:** Health inequalities, health status, autism, transition, life course epidemiology, longitudinal studies

## Abstract

**Objective:** Transition from school to early adulthood incurs many changes and may be associated with deterioration in general health in youth with autism. We aimed to investigate this.

**Method:** The National Longitudinal Transitions Study-2 is a USA nationally representative sample of youth receiving special education services, aged 13–17 at wave 1, followed-up over 10 years in five data collection waves. We conducted random-effects ordered logistic regressions to determine the odds ratios (OR) with 95% confidence intervals of wave, age, sex, ethnicity/race, additional intellectual disabilities, parental/guardian relationship status, and household income being associated with general health status in youth with autism.

**Results:** Across waves, only between 74.3%–69.6% had excellent/very good health (71.7%–58.8% in those with co-occurring intellectual disabilities), but wave was not associated with health status. Associations were with age OR = 1.18 (1.04, 1.33), co-occurring intellectual disabilities OR = 1.56 (1.00, 2.44), and household income OR = 0.61 (0.40, 0.94) at $30,001–$50,000, OR = 0.44 (0.27, 0.72) at $50,001–$70,000, and OR = 0.34 (0.20, 0.56) at $70,001+. Sex, ethnicity/race, and parental/guardian relationship status were not associated with health status.

**Conclusion:** There was little change in general health status longitudinally across the transitional period, but the proportion with excellent/very good health was low at each wave. Transitional planning should consider co-occurring intellectual disabilities, and the wider socioeconomic context in which children/youth with autism are raised. Lack of other longitudinal studies indicates a need for replication.

## Introduction

Youth with autism experience additional health conditions. However, research on their general health status is limited, as most studies investigate specific mental (Hossain *et al.* 2020) and physical (Rydzewska *et al.* 2021) health conditions, such as anxiety (Van Steensel *et al.* 2011), depression (Wigham *et al.* 2017), sensory impairments and physical disabilities (Rydzewska *et al.* 2019b). General health ratings are important as they are associated with morbidity and mortality in the general population (Mewton and Andrews 2013, Young *et al.* 2010). General population studies show a strongly predictive linear change across general health status, with poorer health associated with a higher number of medical appointments and hospital admissions, and increased mortality (Miilunpalo *et al.* 1997, Burnstrom and Fredlund 2001, Heistaro *et al.* 2001, Schnittker and Bacak 2014). General health status is, therefore, commonly investigated in general population but has been little studied in people with autism.

Transition is defined as the process of moving from childhood to adulthood, occurring between the ages of 13 and 25 years. This age range incorporates the period before secondary school exit, and the period of ‘emerging adulthood’ (Arnett 2014). For youth with autism, transition to adulthood may be a time that is associated with the emergence of mental, behavioural, and physical health problems (Bennett *et al.* 2018); hence, studying general health status before, during and after the transitional period from youth to adulthood is particularly warranted (Young-Southward *et al.* 2017a).

We identified only two previous general health studies in youth with autism, and neither were longitudinal across the transition period, so this is a major gap in the literature. A large study using 2011–2012 National Survey of Children’s Health, USA data, identified 1188/56,746 children/youth with autism under 18, and reported lower log odds of good health (−1.30, *p* < 0.001) compared to other children (Rigles 2017). Children/youth with autism also experienced more adverse childhood events than their peers, which were negatively associated with their health (Rigles 2017). Secondary analysis of Scotland’s Census, 2011 identified 25,063/1,548,819 (1.6%) children/youth with autism under the age of 24, and reported fair/bad/very bad general health in 20.0% aged 0–15 years, and 23.5% aged 16–24 years (Rydzewska *et al.* 2019a). Fair/bad/very bad health was associated with female sex (odds ratio (OR) = 1.635), and adolescent age (OR = 1.206) (Rydzewska *et al.* 2019a). Both studies are limited by cross-sectional design.

Youth with autism commonly have intellectual disabilities; a recently reported prevalence in a large study was 18.1% (Rydzewska *et al.* 2019b). Older studies suggested a higher prevalence (Fombonne 2003), with the change likely to be due to broadening of diagnostic criteria for autism to a spectrum disorder, and more recently, greater awareness of autism in people with average or above average intelligence . General health status is poor in youth with intellectual disabilities, especially for females (Hughes-McCormack *et al.* 2018). Youth with autism and co-occurring intellectual disabilities also have bad health, especially females (Dunn *et al.* 2019). However, we did not identify any studies investigating whether general health status of youth with autism differs between those with and without co-occurring intellectual disabilities. As intellectual disabilities are common in youth with autism, it is important to report on health of youth with autism with and without co-occurring intellectual disabilities separately where possible, as well as together.

Using secondary data analysis of the National Longitudinal Transition Study-2 dataset, this study aimed to investigate general health status of youth with autism with and without intellectual disabilities longitudinally for 10 years over the transitional period, and to quantify the extent to which personal characteristics, parental relationship status, and household income were associated with worse health over this period. We hypothesised that general health status would decline over time, and that co-existing intellectual disabilities, female sex, ethnicity/race, parental relationship status, and lower household income would be associated with worse health in emerging adulthood.

## Method

### Study context

The Individuals with Disabilities Education Act (IDEA) is a USA federal law that authorises special education for children with disabilities. IDEA requires states to provide special education and related services consistent with federal standards as a condition of receiving federal funds. Students with disabilities are entitled to receive special educational services through their local school district from the age of 3 to 18 or 21. To qualify, a student must demonstrate a disability in one of the 13 specific categories, one of which is ‘autism spectrum disorder’. Goals in the Individual Education Plan include academic skills, self-care, social skills, physical, speech, and vocational training (Lipkin and Okamoto 2015).

### Dataset

The National Longitudinal Transition Study-2 (NLTS2) follows up an original National Longitudinal Transition Study funded by the US Department of Education. It provides a picture of a nationally representative sample of youth receiving special education services in USA under the IDEA, as they transition to adulthood.

NLTS2 includes five waves of data collection, beginning in school year 2000/2001 (wave 1) when participants were 13–17 and in grade 7 or above. Data collection was then repeated in school year 2002/2003 (wave 2), 2004/2005 (wave 3), 2006/2007 (wave 4) and ended in 2008/2009 (wave 5), when participants were 21–25. Data were collected through various sources, including parent/young person phone interview or mail survey, school survey, and young person assessment, including youth characteristics, and family circumstances.

### Variables

Individuals with autism were identified from a question from parent phone interview and/or mail survey: ‘[YOUTH] is included in this study because [his/her] school or school district indicated at the beginning of the 2000 school year that [he/she] may have received special education services and had an Individual Education Program. With what physical, sensory, learning or other disabilities or problems has [YOUTH] been diagnosed? Code all that apply’. Respondents answered to each of the 22 response options: (1) has no problem/disability/not getting special services, (2) asthma, (3) attention deficit disorder/attention deficit hyperactivity disorder (ADD/ADHD), (4) autism or Asperger’s, (5) (blindness) complete blindness, (6) cerebral palsy, (7) deafness, (8) deafness and blindness, (9) Down syndrome, (10) dyslexia, (11) emotional disturbance/behaviour disorder, (12) hard of hearing/hearing impairment, (13) health impairment (specify disease), (14) learning disability, (15) mental retardation, (16) physical or orthopaedic impairment, (17) speech impairment/communication impairment, (18) spina bifida, (19) traumatic brain injury, (20) visual impairment/partial sight, (21) developmental delay, (22) other (specify).

Youth with autism were identified from option 4, and youth with intellectual disabilities from option 15. Responses to option 21 were excluded from this study since this term is normally used in USA for young children under 5 and is not synonymous with intellectual disabilities.

Parent-rated health ratings of youth with, and without, autism were obtained from responses to: ‘Would you say [his/her] general health is: (1) excellent, (2) very good, (3) good, (4) fair, (5) poor’.

Ethnicity/race data were obtained from responses to two questions and combined into one variable during the original data coding process. The first question read as follows: ‘Please choose one or more categories that best describe [YOUTH’s] race’: (1) White, (2) African American or Black, (3) American Indian or Alaska Native, (4) Asian, (5) Native Hawaiian, or Other Pacific Islander, (6) other (specify). The second question asked: ‘Is [YOUTH] of Hispanic, Latino, or other Spanish origin?’: (1) yes, (2) no, (3) don’t know, (4) refused. During the original data coding, categories 4 and 5 from the first question were collapsed into ‘Asian/Other Pacific Islander’ and a multi-racial background was coded as ‘Multi/Other’.

Marital status of parent/legal guardian was obtained from responses to: ‘Are you/is she/is he/are they/are [YOUTH’s parents]? (1) married, (2) in a marriage-like relationship, (3) divorced, (4) separated, (5) widowed, (6) single, never married, or (7) other’. We collapsed these categories to married/in a marriage like relationship, divorced/separated/widowed, single/never married, and other.

Household income was obtained from responses to: ‘Please tell me which group best describes the total income of all persons in your household in the last tax year, including salaries or other earnings, money from public assistance, retirement, and so on, for all household members, before taxes: (1) $5000 or less, (2) $5001–$10,000, (3) $10,001–$15,000, (4) $15,001–$20,000, (5) $20,001–$25,000, (6) $25,001–$30,000, (7) $30,001–$35,000, (8) $35,001–$40,000, (9) $40,001–$45,000, (10) $45,001–$50,000, (11) $50,001–$55,000, (12) $55,001–$60,000, (13) $60,001–$65,000, (14) $65,001–$70,000, (15) $70,001–$75,000, or (16) over $75,000?’. We collapsed these categories to $10,000 or less, $10,001–$30,000, $30,001–$50,000, $50,001–$70,000 and $70,001 or more.

### Data analysis

We summarised the numbers and percentages of youth with autism with and without intellectual disabilities, and their sex, ethnicity/race, comorbidities, family income, and parental/guardian relationships at wave 1. We then summarised the number and percentage of youth with autism with and without intellectual disabilities reporting excellent, very good, good, fair, and poor health across all waves of data collection in order to investigate trends in general health status over the transitional period. We also identified youth at wave 1 who had general health status recorded at all five waves of data collection, and plotted changes in general health status across the developmental period for this group.

We investigated whether wave of data collection, age, sex, ethnicity/race, intellectual disabilities, parental/guardian relationship status, and household income were associated with general health status (excellent, very good, good, fair, or poor) using random-effects ordered logistic regression models to adjust for correlations between observations repeated on the same people across different waves (Twisk 2013). In order to further investigate whether the association between co-existing intellectual disabilities and health status changed over the transition period, we included an interaction term between intellectual disabilities and wave and intended to perform subgroup analyses if significant. All analyses were conducted in STATA software version MP 16.1.

Where data were missing on a record of autism or intellectual disabilities at subsequent waves, we imputed the record from wave 1 where data were available for all 1019 observations. Information on age was missing for 1 observation at wave 1 where we imputed the middle value of 15 years old. For the remaining waves, we imputed the missing data on age using the formula of ‘age at wave 1 + 2’ for age at wave 2, ‘age at wave 1 + 4’ for age at wave 3, ‘age at wave 1 + 6’ for age at wave 4 and ‘age at wave 1 + 8’ for age at wave 5, as each wave of data collection was conducted with a two-year interval. Data on sex and ethnicity/race were available for all 1019 observations at wave 1, so for waves 2–5 we imputed the records from wave 1. Information on parental marital status had 30 missing records at wave 1, so we randomly assigned one of the four categories of parental marital status. For waves 2 and 3, we set missing marital status as recorded at the previous wave. For waves 4 and wave 5, data on marital status were missing entirely, so we imputed the data as recorded at wave 3. Information on household income and health was imputed using multiple imputation by chained equations (MICE) in the mi package in STATA. We used MICE for health status and household income only because these two variables were the only ones where the values could potentially change over time, i.e. autism, intellectual disability, sex, and ethnicity/race do not change across waves and age was incremented by 2 given the regular intervals in the data collection process. Marital status could not be imputed using MICE because data were completely missing for waves 4 and 5 for this variable (Appendix 1).

### Approval

Approval to access and analyse data was granted by the Institute of Education Sciences of the United States Department of Education (License number: 16090007).

## Results

At wave 1, data on autism were available on 9008/9576 (94.1%) youth; at wave 2, for 6722/9576 (70.2%); at wave 3, for 5532/9576 (57.8%); at wave 4, for 5465/9576 (57.1%); and at wave 5, for 5300/9576 (55.3%) youth. At wave 1, 1019/9008 (11.3%) youth were recorded to have autism of whom 133/1019 (13.1%) additionally had intellectual disabilities; at wave 2, 839/6722 (12.5%) of whom 98/839 (11.7%) additionally had intellectual disabilities; at wave 3, 726/5532 (13.1%) of whom 88/726 (12.1%) additionally had intellectual disabilities; at wave 4, 751/5465 (13.7%) of whom 97/751 (12.9%) additionally had intellectual disabilities; and at wave 5, 727/5300 (13.7%) of whom 91/727 (12.5%) additionally had intellectual disabilities.

### Participant characteristics

Table 1 shows sex, ethnicity/race, comorbidities, parental/guardian relationship, and family income at wave 1, for all youth with autism, and separately for those with and without intellectual disabilities. As expected, there was a higher proportion of males than females. This difference was slightly less pronounced in the group with intellectual disabilities. A higher proportion of youth with autism without intellectual disabilities was white, while in the group with intellectual disabilities, a higher proportion was Hispanic, Latino or Spanish. Youth with autism and intellectual disabilities had higher rates of all comorbidities except asthma, traumatic brain injury, emotional disturbance or behaviour disorder, dyslexia, specific learning difficulties, and ADD/ADHD than did youth with autism without intellectual disabilities. There were no pronounced differences in parental relationship status between the two groups, but youth with autism without intellectual disabilities were more likely to have a higher total household income, than youth with autism and co-occurring intellectual disabilities.

**Table 1.** Demographic characteristics of young people with autism with and without intellectual disabilities at wave 1.

	All youth with autism *N* = 1019 (100%)	Youth with autism and intellectual disabilities *N* = 133 (100%)	Youth with autism without intellectual disabilities *N* = 886 (100%)
*Sex*			
Male	847 (83.1%)	103 (77.4%)	744 (84.0%)
Female	172 (16.9%)	30 (22.6%)	142 (16.0%)
*Ethnicity/race*			
White	616 (60.5%)	72 (54.1%)	544 (61.4%)
African American	243 (23.8%)	32 (24.1%)	211 (23.8%)
Hispanic, Latino or Spanish	123 (12.1%)	23 (17.3%)	100 (11.3%)
Asian/Pacific Islander	32 (3.1%)	5 (3.8%)	27 (3.0%)
American Indian/Alaska Native	5 (0.5%)	^a^	
Multi/other			
*Comorbidities*			
Blindness	21 (2.1%)	8 (6.0%)	13 (1.5%)
Visual impairment/partial sight loss	40 (3.9%)	12 (9.0%)	28 (3.2%)
Deafness	4 (0.4%)	^a^	
Hard of hearing/hearing impairment	20 (2.0%)	9 (6.8%)	11 (1.2%)
Deafness and blindness	3 (0.3%)	^a^	
Asthma	16 (1.6%)	^a^	
Cerebral palsy	35 (3.4%)	12 (9.0%)	23 (2.6%)
Spina bifida	0 (0.0%)	0 (0.0%)	0 (0.0%)
Traumatic brain injury	4 (0.4%)	^a^	
Health impairment	369 (36.2%)	54 (40.6%)	315 (35.6%)
Physical or orthopaedic impairment	60 (5.9%)	18 (13.5%)	42 (4.7%)
Emotional disturbance or behaviour disorder	67 (6.6%)	7 (5.3%)	60 (6.8%)
Speech/communication impairment	131 (12.9%)	18 (13.5%)	113 (12.8%)
Dyslexia	3 (0.3%)	^a^	
Specific learning difficulties	94 (9.2%)	9 (6.8%)	85 (9.6%)
ADD/ADHD	331 (32.5%)	41 (30.8%)	290 (32.7%)
Multiple disabilities	6 (0.6%)	^a^	
Other	179 (17.6%)	30 (22.6%)	149 (16.8%)
*Marital status of parent/legal guardian* ^b^			
Married/in a marriage like relationship	705 (71.3%)	93 (72.1%)	612 (71.2%)
Divorced/separated/widowed	213 (21.5%)	28 (21.7%)	185 (21.5%)
Single/never married	71 (7.2%)	8 (6.2%)	63 (7.3%)
Other	0 (0.0%)	0 (0.0%)	0 (0.0%)
*Total household income* ^c^			
$10,000 or less	63 (7.3%)	10 (8.8%)	53 (7.1%)
$10,001–$30,000	203 (23.5%)	31 (27.4%)	172 (23.0%)
$30,001–$50,000	186 (21.6%)	20 (17.7%)	166 (22.2%)
$50,001–$70,000	165 (19.1%)	25 (22.1%)	140 (18.7%)
$70,001 or more	245 (28.4%)	27 (23.9%)	218 (29.1%)

^a^ Cells combined to avoid statistical disclosure.

^b^ Nine hundred and eighty-nine responses for all youth with autism (30 responses missing), 129 for youth with autism and intellectual disabilities (4 responses missing) and 860 for youth with autism without intellectual disabilities (26 responses missing).

^c^Eight hundred and sixty-two responses for all youth with autism (157 responses missing), 113 for youth with autism and intellectual disabilities (20 responses missing) and 749 for youth with autism without intellectual disabilities (137 responses missing).

### Parent-rated general health status

The proportion of youth with autism who had excellent/very good health was low given their young age. When separated into youth with and without intellectual disabilities, this trend was more pronounced in the group with co-occurring intellectual disabilities. For all youth with autism, across waves 1–5, there was a small change in the proportion reporting excellent/very good health, with fewer doing so with increasing age over the transition period, from 72.7% at 13–17 years to 69.6% at 21–25 years. This trend was influenced more by health ratings reported for the group with co-occurring intellectual disabilities, which were overall lower. Regarding excellent health specifically, for all youth with autism, 40.6% reported excellent health at 13–17 years, declining to 31.9% at 21–25 years. Among youth with autism and co-occurring intellectual disabilities, 30.7% reported excellent health at 13–17 years, declining to 27.4% at 21–25 years (Table 2).

**Table 2.** General health status of young people with autism with and without intellectual disabilities.

General health status	Wave 1 Aged 13–16 years	Wave 2 Aged 15–19 years	Wave 3 Aged 17–21 years	Wave 4 Aged 19–23 years	Wave 5 Aged 21–25 years
*All youth with autism*					
Excellent	404 (40.6%)	340 (42.1%)	273 (39.9%)	235 (36.8%)	198 (31.9%)
Very good	319 (32.1%)	260 (32.2%)	221 (32.3%)	216 (33.8%)	234 (37.7%)
Good	204 (20.5%)	156 (19.3%)	150 (21.9%)	142 (22.2%)	144 (23.2%)
Fair	61 (6.1%)	42 (5.2%)	33 (4.8%)	42 (6.6%)	40 (6.4%)
Poor	7 (0.7%)	9 (1.1%)	7 (1.0%)	4 (0.6%)	5 (0.8%)
Excellent/very good combined	723 (72.7%)	600 (74.3%)	494 (72.2%)	451 (70.6%)	432 (69.6%)
Total	995 (100.0%)	807 (100.0%)	684 (100.0%)	639 (100.0%)	621 (100.0%)
*Youth with autism and intellectual disabilities*					
Excellent	39 (30.7%)	31 (32.3%)	28 (32.9%)	25 (29.4%)	23 (27.4%)
Very good	50 (39.4%)	37 (38.5%)	33 (38.8%)	25 (29.4%)	33 (39.3%)
Good	28 (22.0%)	24 (25.0%)	17 (20.0%)	26 (30.6%)	18 (21.4%)
Fair	7 (5.5%)	4 (4.2%)^a^	7 (8.2%)^a^	9 (10.6%)^a^	10 (11.9%)^a^
Poor	3 (2.4%)				
Excellent/very good combined	89 (70.1%)	68 (70.8%)	61 (71.7%)	50 (58.8%)	56 (66.7%)
Total	127 (100.0%)	96 (100.0%)	85 (100.0%)	85 (100.0%)	84 (100.0%)
*Youth with autism without intellectual disabilities*					
Excellent	365 (42.1%)	309 (43.5%)	245 (40.9%)	210 (37.9%)	175 (32.6%)
Very good	269 (31.0%)	223 (31.4%)	188 (31.4%)	191 (34.5%)	201 (37.4%)
Good	176 (20.3%)	132 (18.6%)	133 (22.2%)	116 (20.9%)	126 (23.5%)
Fair	54 (6.2%)	39 (5.5%)	27 (4.5%)	34 (6.1%)	31 (5.8%)
Poor	4 (0.5%)	8 (1.1%)	6 (1.0%)	3 (0.5%)	4 (0.7%)
Excellent/very good combined	634 (73.1%)	532 (74.9%)	433 (72.3%)	401 (72.4%)	376 (70.0%)
Total	868 (100%)	711 (100%)	599 (100%)	554 (100%)	537 (100%)

^a^ Cells combined to avoid statistical disclosure.

**Figure 1.**
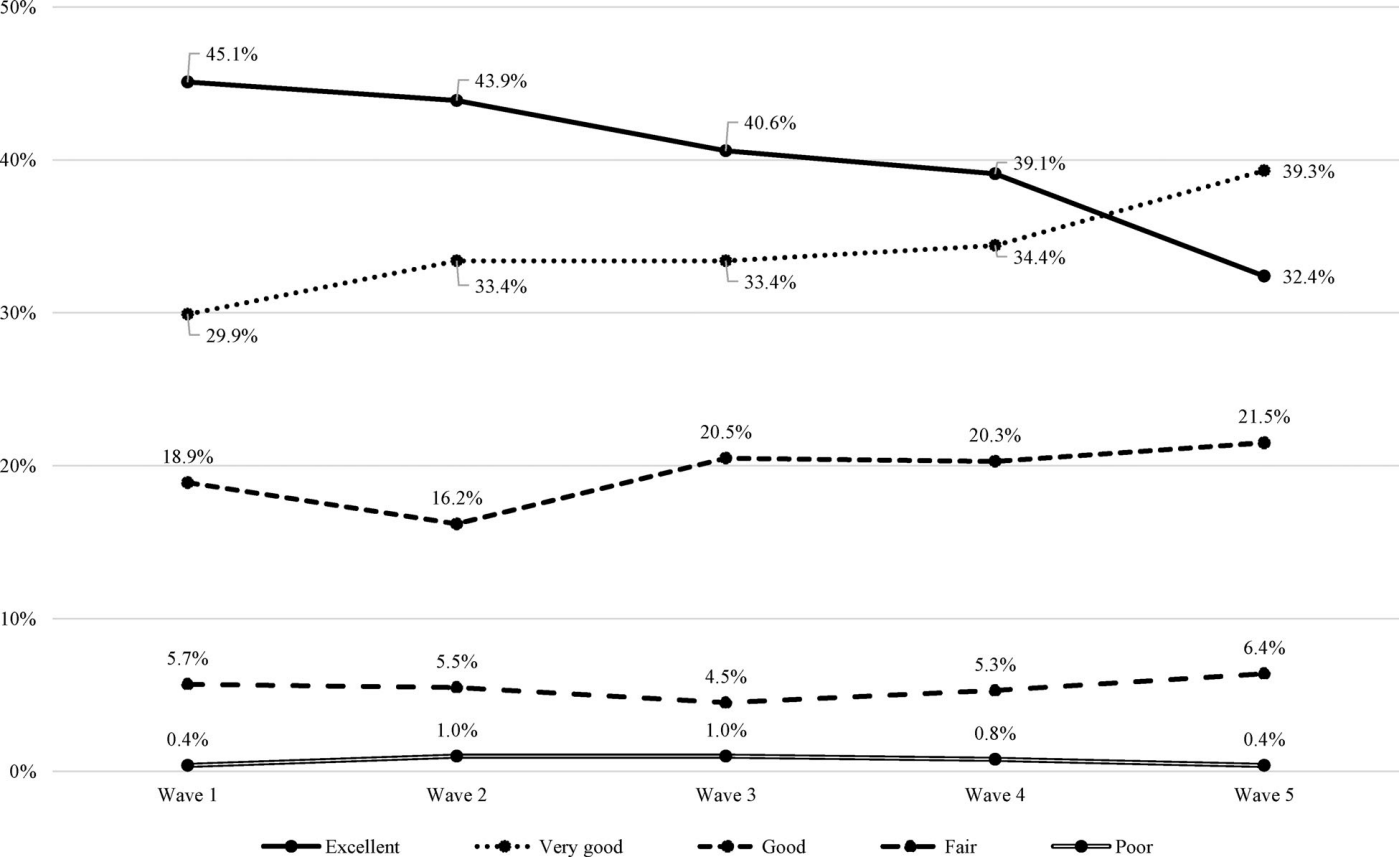
Health of all youths with autism identified at wave 1 and followed across waves 1–5.

Four hundred and eighty-eight young people with autism had a record of parent-rated general health status at all five waves. Figure 1 shows their general health status across the transition period.

Results from a random-effects ordered logistic regression model on all 1019 young people with autism are shown in Table 3. Waves 3–5 were not significantly associated with general health status, and nor were sex, ethnicity/race, and parental/guardian relationship status. Older age groups (OR = 1.176, 95% CI 1.040–1.329) and youth with co-occurring intellectual disabilities (OR = 1.561, 95% CI 1.000–2.436) were one and a half times as likely to experience worse health over this transition period. Higher income was associated with better health, significantly so over $30,001 (OR = 0.612, 95% CI 0.397–0.943 at $30,001–$50,001, to 0.339, 95% CI 0.204–0.564 at $70,001+). When we further investigated whether the association between having/not having intellectual disabilities and health status changed over the transition period, we found that the interaction term between co-occurring intellectual disabilities and wave was not statistically significant.

**Table 3.** The longitudinal effect of individual waves, age, sex, intellectual disabilities, ethnicity/race, parental/guardian relationship status, and household income in predicting health in the whole population with autism.

Variable	Odds ratio	Std. err.	*t*	*P* > |*t*|	95% CI
*Wave*					
1	Ref	Ref	Ref	Ref	Ref
2	0.684	0.151	−2.52	0.012	0.509–0.919
3	0.620	0.251	−1.90	0.058	0.379–1.016
4	0.500	0.393	−1.76	0.079	0.231–1.084
5	0.505	0.508	−1.35	0.180	0.186–1.372
*Age*	1.176	0.062	2.60	0.010	1.040–1.329
*Sex*					
Male	Ref	Ref	Ref	Ref	Ref
Female	1.082	0.218	0.36	0.718	0.705–1.660
*Intellectual disabilities*					
No	Ref	Ref	Ref	Ref	Ref
Yes	1.561	0.227	1.96	0.050	1.000–2.436
*Ethnicity/race*					
White	Ref	Ref	Ref	Ref	Ref
African American	1.117	0.193	0.57	0.566	0.764–1.633
Hispanic, Latino or Spanish	0.963	0.267	−0.14	0.888	0.569–1.629
Asian/Pacific Islander	1.438	0.389	0.93	0.351	0.670–3.086
American Indian/Alaska Native	0.339	1.307	−0.83	0.410	0.025–4.541
Multi/other ethnicity/race	0.084	1.450	−1.71	0.088	0.005–1.445
*Marital status of parent/legal guardian*					
Married/in a marriage like relationship	Ref	Ref	Ref	Ref	Ref
Divorced/separated/widowed	1.306	0.195	1.37	0.172	0.891–1.916
Single/never married	1.305	0.325	0.82	0.413	0.688–2.476
Other parental relationship status	2.171	0.688	1.13	0.262	0.555–8.497
*Total household income*					
$10,000 or less	Ref	Ref	Ref	Ref	Ref
$10,001–$30,000	0.768	0.199	−1.32	0.189	0.517–1.141
$30,001–$50,000	0.612	0.218	−2.25	0.026	0.397–0.943
$50,001–$70,000	0.439	0.249	−3.31	0.001	0.268–0.720
$70,001 or more	0.339	0.257	−4.21	0.000	0.204–0.564

## Discussion

### Summary of principal findings

Contrary to our expectations, longitudinally, an ordinal measure of general health status did not decline over this transitional period. Whilst none of the last ‘waves’ of data collection were associated with general health status across the 10-year period, older age was associated with worse health. Our hypotheses were correct that youth with autism had worse health if they also had intellectual disabilities, and/or had lower household income. However, we did not find any difference regarding sex, ethnicity/race, nor parental relationship status. The proportion of youth with autism reported to have excellent health appeared to decline over the time period overall, and separately for those with and without additional intellectual disabilities.

It is unclear why transition was not statistically associated with decline in the general health status. The study examined parents’ reports of their children’s health, and one possibility might be that parents are more strongly focussed on managing needs associated with autism. Self-reports of quality of life from adolescents with autism have been shown to be more highly correlated with parental proxy-report (i.e. parents’ reports of how they think their child would respond to questions) than with parents’ own report (i.e. parental reports of how they evaluate their child’s quality of life) (Sheldrick *et al.* 2012, Shipman *et al.* 2011). Similar findings have been reported for adults with autism (Hong *et al.* 2016) where self-report of subjective quality of life was more congruent with maternal proxy-report than with maternal report, but there were no significant mean differences between adult self-report and maternal proxy-report in any of the four domains of the health-related quality of life.

Given the high rates of mental (Hossain *et al.* 2020) and physical (Rydzewska *et al.* 2021) health conditions and premature mortality in autism, especially in groups with co-occurring intellectual disabilities (e.g., Hirvikoski *et al.* 2016, Hwang *et al.* 2019, Jokiranta-Olkoniemi *et al.* 2021), the many changes from child to adult health, social, and support services that young people experience over this period, and the low proportion who had excellent/very good general health in this study, it is clearly important to specifically plan around the health needs of youth with autism.

### Comparison with existing literature

We are unaware of any other longitudinal studies of general health status in youth with autism over the transition period with which to draw comparisons. Comparison with cross-sectional studies shows that the USA rates of fair/poor health we report for youth with autism (7% across waves) are lower than previously reported for children (20.0%) and youth (23.5%) in Scotland (Rydzewska *et al.* 2019a). Similarly, our rates of fair/poor health for youth with autism and co-occurring intellectual disabilities are lower than previously reported for children (52.4%) and adults aged 16–65+ (47.6%) in Scotland (Dunn *et al.* 2019). Contrary to our USA findings, these Scottish studies reported fair/bad/very bad health occurring more for females with autism. Our finding that health worsened with increasing age is similar to the cross-sectional study in Scotland (Rydzewska *et al.* 2019a). One study reported that adverse childhood events experienced by children with autism were negatively associated with health (Rigles 2017). We specifically investigated parental/guardian relationship, and household income, finding that the latter was highly relevant, but not the former.

### Strengths and limitations

NLTS2 provides large scale, 10-year longitudinal information on pupils with disabilities from a nationally representative sample of young people receiving special education services, as they transition to early adulthood. Findings should be generalisable to other youth with autism in high income countries. Data on general health provided subjective reports rather than objective measurements. Data used proxy-reports of general health. Without proxy-reporting, we would have no information on youth unable to self-report due to autism-related communication difficulties and/or co-occurring intellectual disabilities, and proxy-reporting is the basis for much of the healthcare provided for people with autism who cannot self-report. Causes of general inaccuracies have been described in both self and proxy-reports on general health in older adults, people with Alzheimer’s disease, and people with intellectual disabilities, with the conclusion that overall, proxy-reports are useful (Perkins 2007). Recently published studies of other datasets also used self/proxy-reported general health status in children/youth with autism (Rydzewska *et al.* 2019a), autism and co-occurring intellectual disabilities (Dunn *et al.* 2019) and intellectual disabilities (Hughes-McCormack *et al.* 2018, Emerson and Hatton 2007a, 2007b, Young-Southward *et al.* 2017b). By design, the NLTS2 study did not include a comparison group of youth without special educational needs.

### Implications

Whilst general health of youth with autism changed little over the transitional period, the proportion with excellent/very good health was low across the 10-year period, especially for those with additional intellectual disabilities, and/or low household income. This highlights the need for services across all ages. This may present particular challenges at transition due to many changes experienced, e.g., leaving school and the supports schools offer, finding employment and day-time activities, and transitioning from paediatric health care services. We have found that older age, co-occurring intellectual disabilities, and lower household income may also negatively impact on young people’s health, highlighting the need to plan support and services which consider additional intellectual disabilities, and the wider family socioeconomic context in which children/youth are raised. There was, however, very little existing research with which we could compare our findings, and no other longitudinal research, suggesting more studies are indicated to replicate our findings.

## Data Availability

Data not available publicly.

## Code availability

STATA code available upon request.

## Appendix 1: Number of observations missing across waves 1–5

**Table:**

Variable	Wave 1 (*n*)	Wave 2 (*n*)	Wave 3 (*n*)	Wave 4 (*n*)	Wave 5 (*n*)
Autism	0	210	315	294	314
Intellectual disabilities	0	210	315	507	314
Age	1	210	315	294	314
Sex	0	210	342	294	1019
Ethnicity/race	0	210	1019	1019	1019
Parental marital status	30	224	353	1019	1019
Household income	157	328	386	441	460
Health	24	212	335	380	398
